# Differences and similarities between disseminated intravascular coagulation and thrombotic microangiopathy

**DOI:** 10.1186/s12959-018-0168-2

**Published:** 2018-07-11

**Authors:** Hideo Wada, Takeshi Matsumoto, Kei Suzuki, Hiroshi Imai, Naoyuki Katayama, Toshiaki Iba, Masanori Matsumoto

**Affiliations:** 1 Department of Molecular and Laboratory Medicine, Mie University Graduate School of Medicine, Tsu, Mie 514-8507 Japan; 2 Division of Blood Transfusion Medicine and Cell Therapy, Mie University Graduate School of Medicine, Tsu, Japan; 3 Emergency Critical Care Center, Mie University Graduate School of Medicine, Tsu, Japan; 4 Department of Hematology and Oncology, Mie University Graduate School of Medicine, Tsu, Japan; 5 Department of Emergency and Disaster Medicine, Juntendo University Graduate School of Medicine, Tokyo, Japan; 6 Department of Blood Transfusion Medicine, Nara Medical University, Nara, Japan

**Keywords:** DIC, TMA, Microvascular thrombosis, Hyperfibrinolysis, Organ failure, Microangiopathic hemolytic anemia

## Abstract

**Introduction:**

Both disseminated intravascular coagulation (DIC) and thrombotic microangiopathy (TMA) cause microvascular thrombosis associated with thrombocytopenia, bleeding tendency and organ failure.

**Reports and discussion:**

The frequency of DIC is higher than that of thrombotic thrombocytopenic purpura (TTP). Many patients with TMA are diagnosed with DIC, but only about 15% of DIC patients are diagnosed with TMA. Hyperfibrinolysis is observed in most patients with DIC, and microangiopathic hemolytic anemia is observed in most patients with TMA. Markedly decreased ADAMTS13 activity, the presence of Shiga-toxin-producing *Escherichia coli* (STEC) and abnormality of the complement system are useful for the diagnosis of TTP, STEC-hemolytic uremic syndrome (HUS)and atypical HUS, respectively. However, there are no specific biomarkers for the diagnosis of DIC.

**Conclusion:**

Although DIC and TMA are similar appearances, all coagulation, fibrinolysis and platelet systems are activated in DIC, and only platelets are markedly activated in TMA.

## Background

Disseminated intravascular coagulation (DIC) [1, 2] is a serious disease that causes microvascular thrombosis associated with thrombocytopenia, a bleeding tendency and organ failure. These symptoms and laboratory data are similar to those of thrombotic microangiopathy (TMA) [3] which includes thrombotic thrombocytopenic purpura (TTP) [4, 5], Shiga-toxin-producing *Escherichia coli* (STEC) - hemolytic uremic syndrome (HUS) [6, 7], complement-mediated TMA (also called atypical HUS; aHUS) [7, 8] and secondary TMA [3, 9]. DIC also has several clinical subtypes, including asymptomatic type, marked bleeding type, organ failure type and complication types such as TTP or heparin-induced thrombocytopenia [10]. As the treatment of DIC [11] differs from that of TMA [4, 12], it is important to perform a differential diagnosis of DIC and TMA. The differences and similarities between DIC and TMA are reviewed in this study.

### Differences in the definition and concept of DIC and TMA

**Fig. 1:**
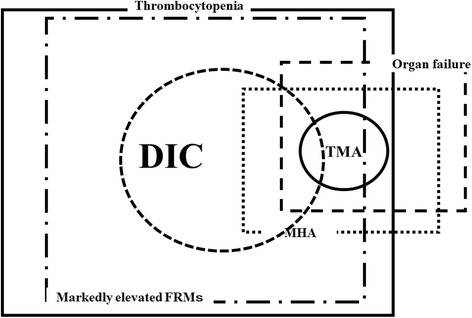
Concept of DIC and TMA. DIC, disseminated intravascular coagulation; TMA, thrombotic microangiopahy; MHA, microangiopathic hemolyitc anemia; FRMs; fibrin related markers

The frequency of pneumonia associated DIC was reported to be about 10,000 cases per year according to the Japanese Diagnosis Procedure Combination (DPC) database [13], suggesting that DIC due to pneumonia occurs in about 70/10^6^ populations. With the addition of other types of DIC, the frequency of all DIC is about 300/10^6^ populations. In contrast, the frequency of TTP was reported to be 2.0/10^6^ populations [3]. These reports suggest that the frequency of DIC in Japan is 150-fold higher than that of TTP (Fig. 1). According to the International Society of Thrombosis and Haemostasis (ISTH), DIC is an acquired syndrome characterized by the intravascular activation of coagulation with the loss of localization arising from different causes. It can originate from and cause damage to the microvasculature, which if sufficiently severe, can produce organ dysfunction. DIC is characterized by the generation of fibrin related markers (FRMs; soluble fibrin monomers, fibrinogen and fibrin degradation products [FDPs], D-dimers, etc.) and reflects an acquired (inflammatory) or non-inflammatory disorder of the microvasculature [1]. Regarding the definition of TMA, TMA presents with microangiopathic hemolytic anemia (MHA), including hemolytic anemia, thrombocytopenia and organ failure in the kidney, central nervous system, and other organs [3, 4]. These findings suggest that marked elevation of FRMs is required in DIC while MHA is required in TMA; the diagnosis of TTP among TMA requires a markedly decreased ADAMTS13 level [14], that of STEC-HUS requires the detection of a STEC infection [15] and that of aHUS requires the detection of abnormalities in the complement system [16].

However, DIC has no specific marker for its diagnosis and is instead diagnosed by a scoring system using global coagulation tests. Furthermore, DIC is often associated with TMA, and TMA is often associated with DIC [17], suggesting that a differential diagnosis between DIC and TMA may be difficult.

DIC associated with TMA was observed in patients with bone marrow metastasis of solid cancer as gastric cancer, those with liver failure and those with group A streptococcal infection. In patients with DIC, bone marrow metastasis mainly causes MHA, liver failure mainly causes an increase in the von Willebrand factor/ADAMTS13 ratio, and group A streptococcal infection mainly cause massive hemolysis. However, it would be much more important to find TMA associated with DIC.

### Differences and similarities in the mechanism of onset for DIC and TMA

**Fig. 2:**
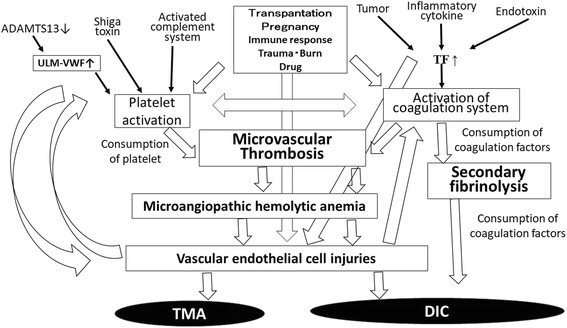
Mechanism underlying onset for DIC or TMA. DIC, disseminated intravascular coagulation; TMA, thrombotic microangiopahy; TF, tissue factor; ULM-VWF, ultra-large multimers of von Willebrand factor

The basic mechanism of onset for DIC is the marked activation and consumption of coagulation system followed by the activation of secondary fibrinolysis [18]. In contrast, the basic mechanism of onset for TMA is the marked activation and consumption of platelets due to several factors followed by the activation and injury of vascular endothelial cells [19, 20] (Fig. 2). Triggers of the activation of coagulation system are reported to include tissue factor (TF) [21, 22], inflammatory cytokines [23, 24] and lipopolysaccharide (LPS) [25], the activation leukocytes [26] and abnormal delivery among others. Trigger of platelet and vascular endothelial cells activation are reported to be a marked decrease in the ADAMTS13 levels in TTP [27], the detection of STEC in STEC-HUS [15] and the detection of abnormalities in the complement system in aHUS [16], along with other factors, such as transplantation, pregnancy, drugs and immune diseases, in secondary TMA [28]. Particularly marked decreases in the ADAMS13 level result in an inability to cleave ultra-large multimers of von Willebrand factor [29, 30], thereby causing platelet aggregation. Markedly fibrinolysis is frequently observed in most patients with DIC, except for some septic DIC cases [31], while markedly fibrinolysis is not observed in patients with TMA.

Both DIC and TMA cause microvascular thrombosis, which is caused mainly by the activation of the coagulation system in DIC and by the activation of platelets and vascular endothelial cells in TMA. Several cases of TMA have been reported to be complicated with hemophilia patients treated with activated prothrombin complex concentrates (APCCs) in the clinical trial for Emicizumab [32]. As APCCs usually causes DIC but not TMA, the differential diagnosis is important in these cases [33]. Although DIC is an acquired disease, Upshaw-Schulman syndrome as familial TTP [34] and many patients with aHUS are examples of congenital TMA.

### Difference in the diagnosis between DIC and TMA

As there is no gold standard for diagnosing DIC and no specific biomarker that clearly diagnoses DIC, the differential diagnosis between DIC and TMA is difficult. Four diagnostic criteria for DIC has been established by the Japanese Ministry of Health, Labor and Welfare [35], ISTH [1], Japanese Association for Acute Medicine [36] and the Japanese Society on Thrombosis and Hemostasis (JSTH) [37, 38]. These diagnostic criteria use a similar scoring system based on global coagulation tests (GLTs) such as the platelet count, prothrombin time (PT), FRMs (Table 1). Therefore, there are no significant differences in the usefulness among various diagnostic criteria for DIC [39]. The diagnosis of TMA is based on the presence of hemolytic anemia (hemoglobin < 10 g/dl), thrombocytopenia (12 × 10^9^/ml) and organ failure. TMA patients with ADAMTS13 < 10%, those with STEC and those with abnormalities in the complement system can be easily diagnosed with TTP, STEC-HUS and aHUS, respectively (Table 2) [4, 40, 41]. However, markedly decreased ADAMTS13 levels have been reported in severe sepsis patients without TTP [42, 43], suggesting that platelet activation due to decreased ADAMTS13 might be observed in DIC patients with severe sepsis. The diagnosis of other TMA aside from DIC with hemolysis is difficult. Most patients with TMA can be diagnosed using several DIC diagnostic criteria to have DIC, but only 10%–15% of DIC patients can be diagnosed to have TMA (Fig. 2).

**Table 1:** Diagnostic criteria for infectious DIC

	ISTH	P	JSTH	P	JMHLW	P	JAAM	P
PLT (× 10^3^/μl)	100 ≧ > 50	1	120 ≧ > 80	1	120 ≧ > 80	1	120 ≧ > 8.0	1
	50 ≧	2	80 ≧ > 50	2	80 ≧ > 50	2		
			50 ≧	3	50 ≧	3	80 ≧	3
Reduction of PLT			30%	1*			30%	1*
							50%	3*
Prothrombin time ratio or Prolongation (s)	3 ≦ < 6.0	1	1.25 ≦ < 1.67	1	1.25 ≦ < 1.67	1	1.2 ≦	1
	6 ≦	2	1.67 ≦	2	1.67 ≦	2		
Fibrinogen (g/L)	1.0 ≧	1			1.5 ≧ > 1.0	1		
					1.0 ≧	2		
Fibrin related markers, FDP (μg/ml)			10 ≦ < 20	1	10 ≦ < 20	1	10 ≦ < 25	1
	Increase	2	20 ≦ < 40	2	20 ≦ < 40	2		
	Markedly increase	3	40 ≦	3	40 ≦	3	25 ≦	3
Antithrombin			< 70%	1				
TAT or SF			2 fold higher of NR					
Underlying diseases					Positive	1		
Bleeding					Positive	1		
OF due to thrombosis					Positive	1		
SIRS							Positive	1
DIC		5 ≦		5 ≦		7 ≦		4 ≦

*ISTH* International Society of Thrombosis and Haemostasis, *JSTH* Japanese Society of Thrombosis and Hemostasis, *JMHLW* Japanese Ministry of Health, Labor and Welfare, *JAAM* Japanese Association for Acute Medicine, *PLT* platelet count, *FDP* fibrinogen and fibrin degradation products, *TAT* thrombin antithrombin complex, *SF* soluble fibrin, *SIRS* systemic inflammatory response syndrome, *DIC* disseminated intravascular coagulation

*PLT and reduction of PLT pointes should be within 3 points

**Table 2:** Diagnostic criteria for TMA [4, 35, 36]

	STEC-HUS	aHUS	TTP	TMA
Hemoglobin (g/dl)	10.0 ≧	10.0 ≧	Hemolysis	10.0 ≧
Platelet (× 10^4^/μl)	15.0 ≧	15.0 ≧	Thrombocytopenia	15.0 ≧
Ogan failure	Renal failure Creatinine ≧1.5 folds of the standard	Renal failure Creatinine ≧1.5 folds of the standard	Neurological symptoms	?
Laboratory finding	Detection of STEC	Genetic abnormality in the complement system	ADAMTS13 < 10%	?

*TMA* thrombotic microangiopathy, *aHUS* atypical hemolytic uremic syndrome, *TTP* thrombotic thrombocytopenic purpura, *STEC* Shiga toxin-producing *Escherichia coli*

### Differences and similarities between DIC and TMA

**Fig. 3:**
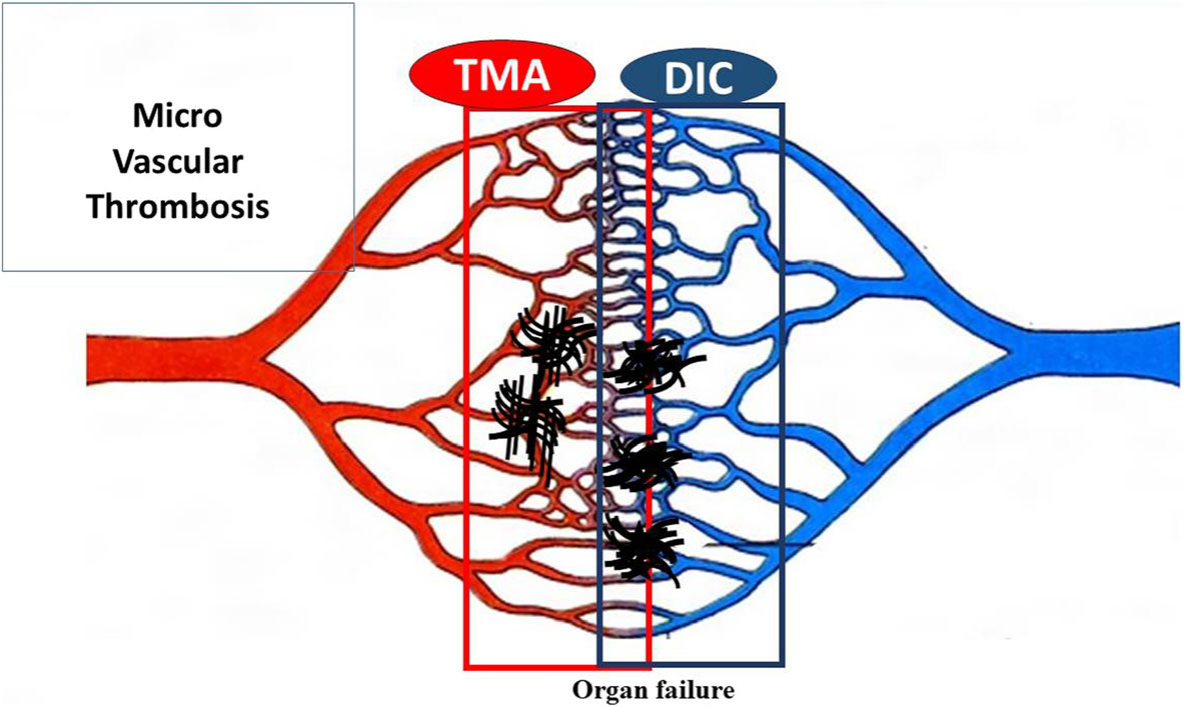
Microvascular thrombosis in DIC and TMA. DIC, disseminated intravascular coagulation; TMA, thrombotic microangiopahy

The differences and similarities between DIC and TMA are described in Table 3. Among clinical symptoms, bleeding and organ failure are frequently observed in patients with DIC as well as those with TMA, but lung or cardiovascular failures is more frequently observed only in patients with DIC [44], while renal or central nervous system failure is more frequently observed in patients with TMA [3]. Hypotension as organ failure is observed in many patients with DIC, while hypertension tend to be observed in patients with TMA [45]. Hypertension may be caused by acute kidney injury or arterial occlusion. Anemia is also more frequently observed in patients with TMA [20, 46] than in those with DIC. Red blood cell fragmentation may be caused by microvascular thrombosis on the arterial side which has a high blood pressure, but not on the venous side (Fig. 3). Among laboratory data, thrombocytopenia is observed in both DIC and TMA. A decreased hemoglobin level and increased levels of creatinine, total bilirubin and LDH are observed in most patients with TMA, but these abnormalities are observed in only 15% of patients with DIC. A prolonged PT and decreased AT and albumin levels are frequently (but not always) observed in patients with DIC. Markedly elevated FRMs are observed in most patients with DIC. As markedly fibrinolysis may dissolve microthromboses in patients with DIC but not in those with TMA, thrombosis of DIC is not usually detected on autopsy. Therefore, elevated FRMs and decreased platelet counts are the most useful markers for DIC [1].

**Table 3:** Differences and similarities between TMA and DIC

		Severe DIC	Severe TMA
Symptoms	Organ failure	Often (Lung, Kidney, Shock)	Usually (Kidney, CNS)
	Bleeding and bleeding tendency	Frequent	Frequent
	Blood pressure	Low	High
	Hematuria	Sometimes	Frequent
	Anemia	Often	Usually
Laboratory data	Platelet count	Low	Low
	Hemoglobin	Often low	Low
	Fibrin related markers	Markedly high	Slightly high
	Prothrombin time	Often prolong	Normal
	Antithrombin	Often low	Normal
	Albumin	Often low	Normal
	Creatinine	Often high	High
	Total bilirubin, LDH	Often high	High
Treatments	Supportive therapy	Recommended	Recommended
	Blood transfusion (RBC, FFP)	Recommended	Recommended,
	Blood transfusion (PC)	Recommended	Not recommended
	Anticoagulant	Recommended (Japan)	Not mentioned
	PE/FFP	Not mentioned	Recommended
	Special treatment	AT, rhTM (Japan)	Hemodialysis (HUS), Eculizumab (aHUS), Rituximab (TTP)

In Japan, regarding the treatment of DIC and TMA, platelet transfusion is contraindicated for TMA [4], while anticoagulant therapy for DIC, but not for TMA, is recommended [10, 11]. Anti-fibrinolytic therapy is recommended for DIC patients with hyperfibrinolysis. Plasma exchange is recommended in most some cases of TMA such as TTP [47], but not for DIC. Antithrombin concentrate [48] and recombinant thrombomodulin [49] for DIC are frequently used in Japan, while eculizumab [50] has proven effective for compliment mediated TMA, such as aHUS, and rituximab [51] is effective for TTP in patients with a high titer of inhibitor for ADAMTS13.

## Conclusion

DIC and TMA are similar appearances, however, all coagulation, fibrinolysis and platelet systems are activated in DIC, and only platelets are markedly activated in TMA. As treatment is different between DIC and TMA, differential diagnosis between DIC and TMA is important.

## Abbreviations

aHUS: atypical HUS
DIC: Disseminated intravascular coagulation
DPC: Diagnosis Procedure Combination
FDP: fibrinogen and fibrin degradation products
FRMs: fibrin related markers
GLTs: global coagulation tests
HUS: Hemolytic uremic syndrome
ISTH: International Society of Thrombosis and Haemostasis
JSTH: Japanese Society on Thrombosis and Hemostasis
MHA: microangiopathic hemolytic anemia
PT: prothrombin time
STEC: Shiga- toxin-producing *Escherichia coli*
TMA: thrombotic microangiopathy
TTP: thrombotic thrombocytopenic purpura
